# 1037. Efficacy of Germinants and Omadacycline for Preventing *Clostridioides difficile* Relapse in a Murine Model

**DOI:** 10.1093/ofid/ofab466.1231

**Published:** 2021-12-04

**Authors:** Noah Budi, Jared Godfrey, Sanjay Shukla, Nasia Safdar, Warren Rose

**Affiliations:** 1 University of Wisconsin School of Pharmacy, Madison, Wisconsin; 2 University of Wisconsin - Madison, Madison, Wisconsin; 3 Marshfield Clinic Research Institute, Marshfield, WI; 4 University of Wisconsin-Madison School of Medicine and Public Health, Madison, Wisconsin; 5 University of Wisconsin-Madison, Madison, Wisconsin

## Abstract

**Background:**

*Clostridioides difficile* is labeled one of five urgent pathogens by the CDC. The urgency is related to the high burden of disease, limited effective antimicrobials, and recurrent *C. difficile* infections (rCDI) from residual spores (Fig. 1). Impervious to antibiotics, *C. difficile* spores could be induced into vegetative cells by germinants, namely taurocholate, for antibiotic targeting. This study aims to evaluate spore reservoir eradication through applying germinants with antibiotics.

Figure 1. Schematic of the infectious life cycle of C. difficile and treatment opportunities

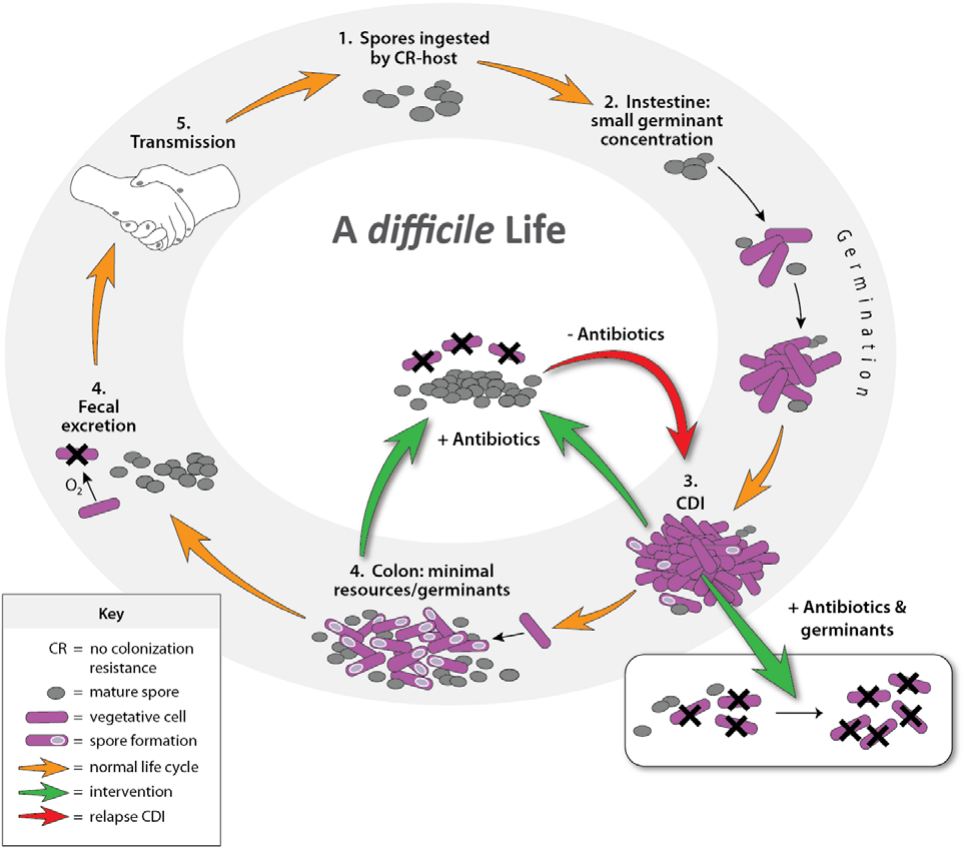

Noah Budi, Nasia Safdar, Warren E Rose, Treatment issues in recurrent Clostridioides difficile infections and the possible role of germinants, FEMS Microbes, Volume 1, Issue 1, September 2020, xtaa001, https://doi.org/10.1093/femsmc/xtaa001

**Methods:**

A published murine model of rCDI using C57BL/6 mice and 1 x 10^5^*C. difficile* spores (VPI 10463) with modification was used (Fig. 2). Six hours after inoculation, mice received 1.5 mg vancomycin (VAN, n=10) or 0.25 mg omadacycline (OMC, n=10) daily by oral gavage until day 4 or either with germinant (G) solution (8 mg of sodium taurocholate, 10 mg of taurine, 0.2 mg of sodium docusate, and 1.72 mg of calcium gluconate) given concomitantly on days 1 to 3 (OMC+G, n=9 and VAN+G, n=8). As a positive control, five mice did not receive antibiotics after spores. To induce rCDI, clindamycin was given on days 10 to 12. Survival, clinical scoring (CS), and weight loss (WL) were recorded until day 15. Fecal samples were taken to measure toxin production and spore shedding. Mice that died prior to day 15, were too sick to provide samples, or had positive stool culture were considered positive for day 15 spore shedding. Fisher’s exact test was used.

Figure 2: Experimental Design

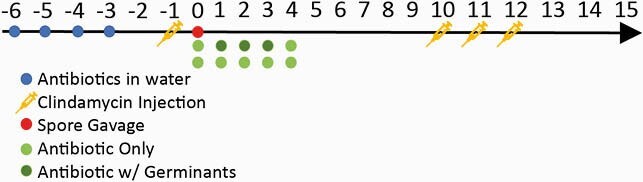

Antibiotic water consisted of kanamycin 0.4 mg/ml, gentamicin 0.035 mg/mL, colistin 850 U/mL, metronidazole 0.215 mg/mL, and vancomycin 0.045 mg/mL given noon to noon on specified days. Clindamycin IP injections given as weight based dose of 10 mg/kg.

**Results:**

Survival is summarized in Figure 3. Both OMC and VAN had 60% survival by day 15 while OMC+G and VAN+G had 100% (p=0.004). Germinant CS and WL were similar to respective antibiotic alone groups until day 8; OMC overall had less severe disease than VAN (Figure 4). Toxin production on day 10 was lower in OMC than VAN, but absent from OMC+G and VAN+G. On day 15, 100% of VAN mice were spore positive compared to 60% with OMC (p=0.087). No mice receiving germinants (OMC+G or VAN+G) were spore positive (p< 0.0001).

Figure 3. Survival Percentage

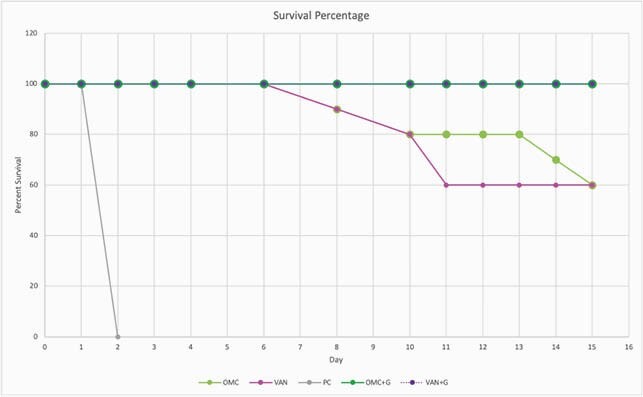

Figure 4. Clinical Scoring and Weight Loss

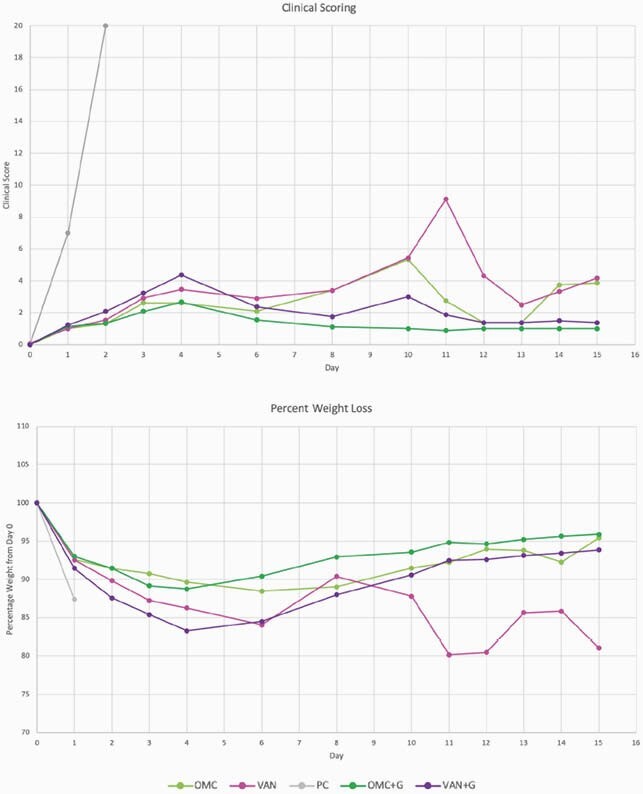

**Conclusion:**

Germinant/antibiotic combinations improved survival in a rCDI mouse model compared to antibiotics alone. Germinants did not induce toxin production when combined with OMC or VAN and eliminated the spore reservoir at the end of treatment. This provides basis for further study of germinants combined with antibiotics to reduce rCDI.

**Disclosures:**

**Warren Rose, PharmD, MPH**, **Merck** (Grant/Research Support)**Paratek** (Grant/Research Support, Advisor or Review Panel member)

